# The Information Flow in a Healthcare Organisation with Integrated Units

**DOI:** 10.5334/ijic.4192

**Published:** 2019-09-26

**Authors:** Åsa Kneck, Maria Flink, Oscar Frykholm, Marie Kirsebom, Mirjam Ekstedt

**Affiliations:** 1 Ersta Sköndal Bräcke University College, Department of Health Care Sciences, Stockholm, SE; 2 Division of Family medicine and Primary care, NVS, Karolinska Institutet, SE; 3 Department of Learning, Informatics, Management and Ethics, Karolinska Institute, Stockholm, SE; 4 Department of Health and Caring Sciences, Faculty of Health and Life Sciences, Växjö, SE

**Keywords:** integrated care, interoperability, information continuity, multimorbidity, patient safety, patient participation

## Abstract

**Introduction:**

Integrated care is believed to provide support to patients with multiple long-term and complex conditions. Transparency in information delivery is key for shared decision-making, and co-production of care. This study aimed to explore information pathways within an integrated healthcare and social care organisation and describe how information continuity was delivered for an older patient with complex care needs.

**Methods:**

An explorative single-case study conducted in a Swedish healthcare organization where municipality and the county council have integrated their services. One focus group discussion and six individual interviews were conducted.

**Results:**

Information flow to partners in care was obstructed, with compensatory double documentation, complementary information channels, and information loss. A heavy burden was on the patient and relatives to keep track of and communicate information between different caregivers. Patients were expected to be active partners in their own care, but were largely excluded from the information flow.

**Discussion:**

Even integration of care organisations does not imply that integrated care is delivered at the sharp end of practice. An integrated electronic health record is needed to improve accessibility of care information from within all the organisations, facilitating handovers between professionals and levels of care, and involving patients in the information flow.

## Introduction

Older people with multi-morbidity are recognised as a particularly vulnerable group, dependent on relatives and an appropriate healthcare service to manage daily life and experience quality of life despite illness. Patients referred to as having complex needs are defined as having three or more chronic conditions affecting three or more body systems; having comorbidity of physical conditions together with depression; being prescribed more than ten drugs; and being housebound or living in a nursing home [1]. These patients often need care services from multiple healthcare providers, and are therefore in need of integrated links that bind the different entities together. Earlier studies have shown that a fragmented healthcare system impacts on older patients with complex needs in particular [2], as they are vulnerable to the information gaps that multiple care transitions may imply [3]. Increasingly, adequate information on self-management after hospital discharge [4] is requested, as patients and their families are expected to take an active role in understanding and managing symptoms and complex drug regimens, including knowing when and where to seek care.

Integrated health service delivery is defined by the WHO as “an approach to strengthen people-centred health systems through the promotion of the comprehensive delivery of quality services across the life-course, designed according to the multidimensional needs of the population and the individual and delivered by a coordinated multidisciplinary team of providers working across settings and levels of care” [5, WHO page 10]. A recent review identified 12 domains and 175 key aspects of integrated care [6], indicating that the delivery of integrated care for people with complex and long-term care needs is an overwhelming task, seemingly hard to fulfil. Integration of care in terms of delivering continuous, comprehensive and coordinated care across organisations and professionals along a patient’s care trajectory is closely related to continuity of care [7], i.e., continuity in management, relations and information [8]. Continuity of care has been associated with reduced utilization of healthcare [9,10] and mortality [9], as well as increased patient satisfaction [10]. One important aspect is information continuity. How information is captured, processed, communicated and applied will affect how the information is received by patients, relatives and the multidisciplinary teams of healthcare professionals. Accessible information and transparent information flow, within care and between all the partners in care, is thus necessary for shared decision-making and responsibility, and co-production of care [11]. A productive interaction between informed patients and experienced and accountable clinicians serves to empower patients as active partners in care [11,12,13,14]. As such, integrated care might contribute towards the triple goal of improving population health, improving individual experiences of care, and reducing per capita costs of care [15].

The Swedish tax-funded healthcare system, with its high specialization, differentiation into three levels of care, and a mixture of both public and private healthcare providers [16], is not well-equipped to meet the challenges of information continuity for a growing and aging population with complex care needs. In Sweden, the central government has the responsibility for legislation, the 21 county councils have the main responsibility for provision of primary healthcare and all branches of specialised in-hospital care. The responsibility for elderly care, care of the functionally disabled, and long-term psychiatric care was transferred from the county councils to the 290 municipalities a few years ago [17]. The county councils and municipalities can largely organise care within their areas of responsibility as they prefer, meaning that different models for care are used across the country. Collaboration between the levels of care are obstructed by both organisational boundaries and governance through different regulations and laws [18]. Laws relating to privacy (e.g., the Personal Data Act), hospital routines, and professional culture have been shown to impede transparency in information transfer between the multidisciplinary providers of out-of-hospital care [19]. The fragmentation of care for a patient lies in that providers are responsible for distinct facets of care, while also working in silos with few cross-diagnostic meeting points, where a holistic view of the patient could otherwise have appeared.

Sweden has used a range of initiatives at a local management level, as well as various legislative acts, to provide continuity of care through enhanced collaboration between hospitals, primary care, and municipalities [20], and to strengthen the patient’s position as an equal partner in own care [21]. Shorter hospital stays and increased demands on patients to be active in own care are discussed as results of an overburdened healthcare system [22], but also as an opportunity for patients to develop competence and shape their daily lives [23]. However, the implementation of integrated chains of care into existing routines is still far from realised, despite some local attempts to do so. In general, these local initiatives focus on extended collaboration between the municipalities and the county council, at both the manager and the provider level.

This study was carried out within a local area, unique in Sweden, where the municipality and the county council have integrated their services within the same organisation [24]. The aim is to create better social care and healthcare for citizens through both horizontal (health services, social services, and other care providers) and vertical (primary, community, hospital, and tertiary care services) integration of care. The organisation is managed as a single company co-financed by the county and the municipality through taxes. This company has been responsible for all levels of care for more than a decade. However, political reforms promoted by new public management have led to outsourcing of services and a growing private market. This means that there are primary healthcare centres and social home care services within the municipality that are not a part of the integrated care organisation. This is well in line with the view that the individual’s freedom of choice is of highest priority [25], but the consequences on functional integration of care and information continuity for persons living with complex health conditions are unclear.

The aim of this study was to explore the information pathways within an integrated healthcare and social care organisation and describe how information continuity was delivered for an older patient with complex care needs.

## Methods

### Design

This study has an explorative qualitative single-case study with a descriptive design, using focus group discussions and individual interviews.

### Description of the integrated care case

The present study was conducted in an integrated healthcare and social service organisation, as described in the introduction, providing hospital care, primary care, and social care to approximately 60,000 citizens in a municipality in mid-Sweden.

### Participants and data collection

Recruitment of participants and data collection for this study was performed during 2016, in two steps. First, participants were recruited for a focus group discussion with a purposive sampling approach to include participants with a diversity of experiences of information transfer and responsibility for different aspects of care for patients over 65 years with multiple chronic illnesses [26]. The participants were recruited by one of the quality developers in the integrated organisation, to represent multiple disciplines and different units of the integrated organisation (specialist in-hospital care, primary healthcare, rehabilitation, and municipal social service and healthcare) (see Table 1). Next, additional people with central functions within the organisation, relevant to the aim of the study, were recruited for participation in individual interviews. All participants in the study had a least 5 years’ experience of working in the current healthcare organisation. The research group had an interdisciplinary composition, including nurses, social workers and an engineer/system developer.

**Table 1:** Participants in the focus group and individual interviews.

Title, profession	Organisation	Focus group	Interview

Physician	Hospital	X	
Pharmacist	Hospital	X	
Head of department, RN	Hospital	X	
RN	Hospital	X	X
Quality developer	Hospital	X	
Quality developer and RN	Hospital	X	X
Administrator	Hospital		X
Occupational therapist	Home care	X	X
Social worker	Municipality	X	
RN	Primary care	X	
RN	Hospital		X
Assistant nurse	Hospital		X

* RN = Registered nurse.

In all, one focus group discussion and six individual interviews were carried out (see Table 1 for information on participants). In the text, the participants are referred to as “healthcare professionals.”

**Figure 1:**
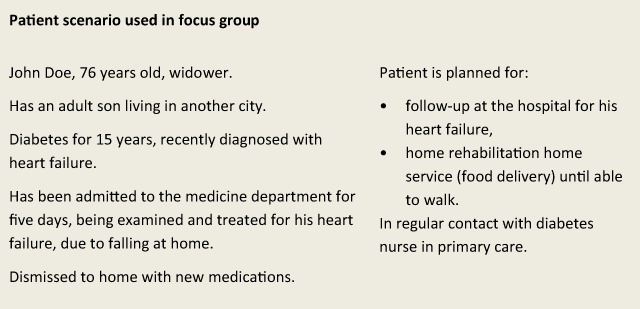
Information flow for a fictitious older patient with chronic illnesses and complex needs.

To get an overall picture of the study topic from the perspective of the healthcare professionals, and to encourage discussions that engaged all participants, a scenario with a fictitious patient was used: an older man with multiple chronic illnesses and complex needs, in transition from hospital care to home care and home rehabilitation (see Figure 1) [27]. Using a fictitious scenario to stimulate participants’ interactions and discussions is consistent with the methodology of focus group interviews [27,28]. The patient journey triggered discussions and all participants contributed with their experience by relating what information they needed in order to be able to care for the fictitious older man – and from whom. Further, the discussions focused on what information they would pass on to the next professional or healthcare provider or to the patient and relatives.

**Figure 2:**
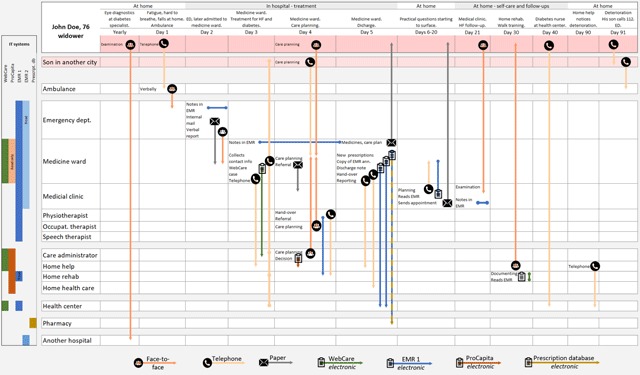
Map of the information flow through different information channels along a fictitious patient’s care trajectory through the healthcare system.

The individual interviews were guided by a semi structured interview protocol based on data from the focus group discussion. Based on the same data, the information pathways within the system were mapped (presented in findings, see Figure 2). This map was used in the individual interviews to deepen and clarify the information pathways and to incite participants to talk about and reflect on the information pathways, as well as the fictitious patient’s possibilities to be involved in own care. The interviews started by identifying the participant’s position in the integrated care system (professional role, function, and placement in the system) followed by mapping the information pathways that the participant used for transfer of information. Specific questions addressed each specialty and position in the system, in order to follow up on issues raised in the focus group discussion and give participants the chance to explain their thoughts and experiences. For example, the professionals at the hospital were asked what information they transferred at discharge and to whom, what information systems they used and if they knew that the information reached the receiver organisation. The professional in home care was asked if they got the information they needed to follow up care for the patient at home and what they did if the information was insufficient.

### Analysis

Data consisted of transcribed texts from the focus group discussion and individual interviews. Qualitative inductive content analysis was used to analyse the “what” at a descriptive level close to the texts, and to describe variations and identify similarities and differences in the texts, as well as descriptive themes that did not vary across the texts [29,30]. The analysis was considered appropriate, as different levels of abstraction could be applied based on the available texts [29,30,31] (see Table 2 for the analytical steps).

**Table 2:** Example of the analysis procedure.

Theme	Coherent information flow is challenged by a lack of interoperability of information systems across the organisation		

**Category**	Isolated EHR systems	Complex health care systems lacking collaboration	Lost information despite major efforts
**Condensed meaning unit**	Calling several numbers for information	Patient referred to several parts of the health care system	Patient makes contact when things do not go as planned.
**Meaning unit**	*I get a number or three numbers to some nurse there and then I have to call until I get the right person…you have to make the rounds*	*Your blood sugar is high – you should talk to the diabetes nurse – and I’m having trouble breathing – you should call the heart failure nurse*	*They call; the home care staff hasn’t shown up, have they got the wrong address, when were the tests supposed to be taken?*

During the analysis process, the text was read through several times, after which “meaning units” composed of words with common contents were extracted from it [29]. As a next step, “condensed meaning units” were formulated, constituting a reduction of the original meaning units without changing the contents of the text. Condensed meaning units with similar contents were then sorted into categories, related to each other and either divided into smaller subcategories or pooled into a broader one. Finally, the cores of the interpreted meanings of the categories were linked together into descriptive themes [29,30] (see Table 3 for categories and themes). The findings are presented as two themes in which the categories are embedded and exemplified through quotations.

**Table 3:** Themes and categories.

Theme	Coherent information flow is challenged by a lack of interoperability of information systems across the organisation	Expected to be an active partner but excluded from the information flow

**Category**	Isolated EHR systems	Home alone
	Complex health care systems lacking collaboration	Determination of diagnose
	Lost information despite major efforts	Negative consequences

## Ethical considerations

The study was approved by the Regional Research Ethics Committee (2014/1498-31:2). Further ethical considerations have been taken into account continuously, throughout the research process. The participants were guaranteed confidentiality, e.g., by ensuring anonymity in quotations used, as well as storing data on participants and interviews in a secure way. Participants were informed of their right to withdraw from the study until data were being analysed, as well as of the fact that their participation was voluntary [32,33].

## Results

The information pathways described by healthcare professionals for a fictitious older patient with multiple chronic illnesses and complex needs encompassed a massive flow of information. A coherent flow of information to different partners in care was obstructed by a lack of interoperability of the electronic information systems, challenging the patient experience of integrated care. Patients were expected to be active partners in their own care, but were largely excluded from the information flow. The patients’ preferences, needs and medical history were difficult to track.

### Coherent information flow is challenged by a lack of interoperability of information systems across the organisation

The main flow of information was between units within the specialist inpatient care and from these units to primary care. Occasional information pathways existed between specialist inpatient care and professionals within social care or home care service; information transfer was most seldom described between healthcare and patients and/or relatives (see Figure 2).

The professionals experienced that information was “locked” within different electronic medical record systems that obstructed information flow. As illustrated in Figure 2, the different healthcare providers involved along a patient’s care trajectory used different electronic systems (see left y axis), and different systems were also used for different purposes within a single organisation. The municipality used one electronic medical record system for documentation, while the hospital and primary healthcare centres used another (i.e., EMR1, Figure 2). The professionals did not always have access to each other’s system, but in some cases had access to read relevant information, in a way that they perceived as unstructured. To facilitate discharge planning and coordinate post-discharge activities between the municipalities, primary healthcare centres and the hospital, a joint web-based communication system was used, which was not compatible with either of the other two systems (i.e., WebCare, Figure 2). In this communication system, the municipality primary healthcare centres and the hospital could share information about a patient’s post-discharge needs and co-create plans for the follow-up activities within primary healthcare and social care (see care planning day 4 and discharge information flow, day 5, Figure 2). The EMR systems at the hospital and primary healthcare centres were not compatible with the systems used in their corresponding specialist hospitals either (EMR2 at the y axis, Figure 2). Consequently, staff caring for the same patient within the integrated healthcare organisation but in different settings (healthcare, social care or rehabilitation) could not get access to all the information concerning their patient, which led to *ad hoc* solutions for communication.

A range of actions were undertaken at the micro-level of the organisation, to ensure that sufficient information kept pace with the patient throughout the chain of care. Professionals used multiple channels of communication as a complement to the digital documentation, to ensure information transfer: face-to-face meetings, telephone and telefax, and handwritten notes distributed as internal mail at certain times of day (see symbols on the x axis, and arrows in Figure 2). Nursing professionals accompanied patients between hospital units to transfer verbal information to the next unit in person, despite there being written information in an electronic medical record, which had also often been communicated by phone at an earlier stage. These compensatory adaptations to the deficient information systems were aimed at ensuring safe and high-quality care as well as creating a prepared practice team. Still, the participants doubted the effectiveness of their procedure.

Coordination of care was described as growing more complex due to an increasing number of patients with multiple illnesses in need of treatment from within multiple specialties, and the legal changes leading to the introduction of competing companies. Services that were previously handled by a single care provider were now offered on an open market, increasing the number of providers involved and hence increasing the risk of information falling through the cracks. For example, food distribution for persons living at home, which was formerly handled by home-help services, was now handled by a food company. The outsourcing led to an increased number of stakeholders and a risk of gaps in food distribution was apparent.

The outsourced transfer service from hospital to home was another example with consequences for patient health and wellbeing.

“When he [the patient] came home, he couldn’t get into the house, he couldn’t get up the stairs, because the wrong kind of transport has been ordered, [the staff] didn’t know that he couldn’t climb stairs. So he had to wait until they had got hold of another transport company that had a stairclimbing device, while he just sat outside.”

Patients, relatives and healthcare professionals all played important individual roles in ensuring that information was passed on between healthcare settings when patients sought care after a period at home. The professionals strived to get enough information to perform their tasks, but some detective work was required to ensure that a patient’s medical history, as well in their narratives about experiences thereof followed into a new healthcare setting. It was not only the deficient information systems (i.e., lacking access to electronic medical records) that hindered information flow. The most essential information was also difficult to find, as all the information was lumped together in an unmanageable mass. Searching for the information needed was described as a common and time-consuming task for healthcare professionals, as well as for patients and their relatives. The professionals said:

“Then he came back to the ER without any kind of documentation and we had to start over from where we left off, right… Because he hadn’t taken what he was given.”

These examples show that the current organisation, despite its goals of care integration, jeopardised the quality of care as well as safety, even when regulations and stated practices were observed.

### Expected to be an active partner – but excluded from the information flow

Professionals were aware that patients at home often lacked appropriate information to manage their multiple illnesses on their own. For instance, patients could not get access to information about their medication regimens (i.e., knowing what to take, how and when), which symptoms might occur, and who to contact for different needs. Although information was verbally communicated to patients during hospital stays, it was difficult for patients to grasp all the information. Thus, information needed to be available also after discharge.

“There’s a lot of sensory input and maybe you’re tired… You could be in pain and so on, so it’s probably common that you come home and don’t really know and maybe come back for that very reason.”

According to the healthcare professionals, patients often turned to emergency care, not primarily due to severely worsening symptoms, but rather because of a lack of information related to self-management, or information about where to turn to get help. Deficient information at discharge turned into more work for the healthcare professionals later, in terms of providing patients with phone numbers to various places and guiding them through the system.

“If they don’t know who they should contact and the ER is always open, but everything else closes in evenings and on weekends… And then they come here, even though they don’t really need to.”

The patients did not have any access to the different information systems, not even the web-based communication system (WebCare) where the planning for discharge was shared between different stakeholders (see for example day 4, Figure 2). Patients and relatives were involved through face-to-face or telephone meetings and their narratives, preferences, and needs were not visible to them, nor could they add any information to the documents. The documentation was transmitted to patients in paper form, at discharge and in the form of referrals to follow-up meetings (see day 5 or “at home” in Figure 2).

The information given at discharge depended on several factors. First, the specific diagnosis of the patient often determined the information given. For some diagnoses, the information pathways were more defined than for others. This was especially evident in cancer care, where dedicated contact nurses were available to support patients with the information needed. Second, specific information was given at certain occasions, for example as a directed information session for a specific group of patients at a “heart school.” If a patient for some reason could not participate, or chose not to, that patient did not receive the information. Third, the information flow was determined by where the hospitalised patient was discharged to. If the patient needed further hospital care, the information from professionals was transferred to other professionals, but this seldom occurred if the patient went home. The fourth factor that determined the information flow was the impact of relatives. The healthcare professionals suggested that patients with determined relatives got better care than others. They described a “relative-dependent care,” where relatives filled gaps when there was a lack of information, leading to unequal treatment of patients. A patient’s network of involved family members played a large role in the patient’s needs being sufficiently met.

The lack of information to patients and their relatives had the consequence that they did not understand that waiting times, for instance, are often due to the complexity of healthcare, not loss of referrals or information. Patients were neither involved in, nor aware of, the number of different procedures that had to take place after a decision on referral, before a patient actually got an appointment. One example showing that patients did not understand how healthcare was organised was that patients often turned to the unit from where the *referral was sent* instead of turning to the unit to which *the referral was addressed*, or another appropriate level of care (i.e., primary care instead of hospital care). Despite the integration of the different care systems, the “wrong” instance could not answer the patient’s questions without taking on additional work to find the information. One professional reflected as follows:

“It is symptomatic of healthcare that everyone thinks the patient should be somewhere else.”

Patients were often asked to participate in care planning and to make decisions about treatment and care, even when they didn’t have adequate information. Most information to patients and relatives was given face-to-face or by phone (Figure 2). According to the informants, patients and their relatives most often got involved in care planning when they thought that there was a problem, when information was missing or when care planning did not proceed as they expected. The healthcare professionals perceived that this was not reasonable:

“So that’s a catch-22. The provider of home-help should be summoned to the care planning meeting, but [we can’t summon them] if we don’t know if the patient will be getting home-help or if [the patient] hasn’t chosen [home-help service provider] yet, as the patient decides that at the care planning meeting when the service assessor has determined that he is eligible for home-help.”

The examples show how the organisation of care affects the quality of care. Even though patients and relatives are invited to participate in decisions and planning of care, this will not achieve its purpose when relevant information is lacking. Gaps in information flow were described due to failure in information technology and inter-organisational governance. The professionals were also aware that both patients and their relatives needed knowledge on how to navigate the system. It was a common conviction among the participants that patients became more dependent on healthcare than necessary due to insufficient and lacking information, but few solutions were suggested.

## Discussion

This study describes the challenges in providing information continuity for patients with complex care needs through their care chain in an integrated healthcare organisation, with a hospital, the surrounding primary healthcare centres, and municipal social care all organised within a single company. This study showed that a structural integration of healthcare and social services at the meso-level (in the organisation) did not result in improved outcomes and experiences of an integration of care processes at the micro-level, where patient care is delivered. A functional interdisciplinary integration [34] is dependent on satisfactory pathways of communication and information transfer between professionals across clinical borders [35,36]. Professionals sharing responsibility, accountability and innovative initiatives to secure information transfer, despite any lack of interoperability across IT systems, is key for providing integrated care [6,11].

In this case study, many current deficiencies in the management of chronic illness were shown, such as deficient care coordination, non-supporting communication systems, and inadequate patient support to manage illnesses. However, a key for successful self-management support and improved outcomes for patients with chronic conditions is a productive interaction between informed activated patients and a prepared proactive practice team [11]. In the current case, a heavy burden of care was placed on the fictitious patient and his relatives, to keep track of, understand, and communicate information from different caregivers, to understand the treatment, and know where to turn in case of questions or worsening symptoms. A patient’s possibility to actively participate in their own care, including monitoring of symptoms, problem-solving, decision-making, and knowing when and where to seek care [37,38,39] was shown to be difficult in this study. The information to patients seemed to be *ad hoc* or dependent on contextual factors. A system for ensuring that patients have received all the necessary information should be inherent in all steps of care, regardless of diagnosis, relatives or where the patient is discharged to. This could help patients become prepared and active in managing their health and facilitate patient navigation of the system [40].

The challenge of communication within complex systems is by no means new. In this study, the lack of a well-functioning clinical information system affected the functional integration of care in terms of unmanageable information masses within units and lack of support for delivery of information across units and to patients and families at home. This made it difficult for the healthcare professionals to become proactive and prepared in their follow-up of patient needs. Paradoxically, there was a massive flow of information through the system, but the information continuity at the sharp end was often dependent on each professional’s “detective work” to get relevant information and/or the ability of an individual patient and/or family caregivers to make information available to various healthcare providers. In Sweden, social care within the municipalities is governed by different legislation than healthcare within primary healthcare and at hospitals. This has further complicated both sharing of and access to information.

Organisation of delivery systems, decision support and clinical information systems are all essential components for improving chronic care management in practice. For the professionals in this study, the macro-level integration of healthcare and social care into the same administration did not facilitate delivery of care on a daily basis. Daily work was still organised in “silos” with few cross-diagnostic meeting points where professionals from different specialties and patients could perform information exchange, shared decision-making, and care planning. The lack of functional integration [34] was largely related to patients’ medical records being “locked” within the separate information systems at each unit, despite the common overarching organisation. The professionals at the sharp end of practice still struggled to get an overview of the information and were challenged in securing information continuity. Patients, on their part, were subject to a multi-faceted struggle: managing their illness, understanding their symptoms and self-management, *and* navigating the system [2,4]. Lessons learned include that meso and macro-level integration of the system, with the theoretical possibility of tracking a patient’s care trajectory, has to be accompanied by functional integration of communication systems and work processes across institutional borders and levels of care. Changing the clinical information systems may take many years. However, improvement in the organisation of care provision may offer more low-hanging fruits to focus on, including team functioning, leadership, and the clinical process of self-management support (e.g., individual care plans and follow-up appointments).

At the macro-level, changes in national regulations promoting competition between companies in the healthcare and social care sector [41] challenged information continuity. For example, the Act on Systems for Freedom of Choice [41] states that patients must choose their service agency provider – as a means of empowerment. Ironically, this has led to catch-22 situations in discharge planning, where nobody has the power to act. Another example at the macro-level was that food distribution, which had previously been handled by home-help services, was now a service purchased from a company, along with transportation. Axelsson and Baihari [42] argue that contractual relations, with organisations competing on a market, are the lowest form of integration. The contractual relationships between transport services, home-help services, and food distribution created additional links in the system which further obstructed information continuity. At a policy macro-level, many values are fighting for space: freedom of choice, autonomy, integrity, market-driven and competition-exposed services, quality, and safety, as well as continuity and person-centred care. Increased privatization and emphasis on patients’ “choice of care,” such as the possibility to choose providers in certain healthcare settings [41], create a diversity of involved parties, which seems to further contribute to the fragmentation of care, impacting on the degree to which care is experienced as integrated. Complex systems [35] like healthcare are never fully knowable, meaning that changes in national regulations may have unforeseeable consequences if other regulations are not synchronised. Lessons learned from the Swedish model are the need for an overall risk-benefit calculation in relation to all the aspects of care that might be affected by introducing new ways of organizing care. The current case highlights a clash between macro-level goals (i.e., freedom of choice and demands from a competitive market) and the micro-perspective goals of a person-centred view on continuity of care, empowering patients to be activated as equal partners in care. It is relevant to question if these two viewpoints can ever meet. Without radical and long-term rethinking of how care processes and information systems are organised, integrated care is just a vision.

Limitations of this study include that only one single integrated healthcare setting was studied, which limits the transferability of finding. The majority of the interviews were conducted with professionals from the hospital setting, and a strength is that both management staff and professionals from different disciplines and from both primary care, specialist care, and municipal care were represented. However, a broader picture would have emerged if we also had included staff from the medical and information technology department. Although the fictitious patient trajectory was based on knowledge drawn from our former studies, i.e., interviews with patients, relatives and observations of patients-physician consultations, involving patients and/or relatives would have strengthened the focus group discussions further. Future studies could add information on the perspectives of patients and relatives on continuity of care in integrated care settings.

## Conclusion and implications

This study confirms that even within a care organisation with a macro-level solution and an explicit aim to provide integrated care, information continuity is challenged by the overarching decisions made at the governmental level. The results from this study suggest that major changes at the micro-, meso- and macro-levels are needed to transform a care system so that integrated care is realised. At the policy level, there is a need for awareness of how changes in policies and laws affect the efforts to achieve integrated care and the interactions at the micro-level. At the healthcare organisational level, there are a number of implications. For example, organisation and distribution of information to all patients is needed, regardless of diagnosis or contextual factors. This might be achieved through proved education methods to support patient activation in self-management and patient understanding of the system. Improving integrated care at the micro-level of the system requires constant adaptations and adjustments to deficiencies in the organisation and communication systems. The electronic medical record systems need improvement in terms of possibilities to summarise what has been accomplished and hand over to the next level of care. Professionals need to be involved in the process of developing the electronic medical record systems. Further accountability for information sharing across fuzzy borders is needed, actively involving patients in the information flow. Information continuity can be managed through recordkeeping and sharing [43], and by providing sufficient information on past events and personal circumstances to the next care provider [8,36]. A key is proactive interaction, emphasizing the patient’s role in managing health using motivational support strategies, including goalsetting, action plans, feedback and follow-up.

## References

[1] Wallace, E, Salisbury, C, Guthrie, B, Lewis, C, Fahey, T and Smith, SM. Managing patients with multimorbidity in primary care. BMJ (Clinical research ed), 2015; 350: h176 DOI: 10.1136/bmj.h176 25646760

[2] Summer Meranius, M. ‘Era delar är min helhet’: En studie om att vara äldre och multisjuk (You see parts but I am whole. A study of older persons’ experience of multimorbidity) Linneaus University Dissertations No 11/2010. ISBN: 978-91-86491-13-0 Written i Swedish with a summary in English; 2010.

[3] Leppin, AL, Gionfriddo, MR, Kessler, M, Brito, JP, Mair, FS, Gallacher, K, et al. Preventing 30-day hospital readmissions: A systematic review and meta-analysis of randomized trials. JAMA internal medicine, 2014; 174(7): 1095–107. DOI: 10.1001/jamainternmed.2014.1608 24820131 PMC4249925

[4] Flink, M and Ekstedt, M. Planning for the Discharge, not for Patient Self-Management at Home – An Observational and Interview Study of Hospital Discharge. International journal of integrated care, 2017; 17(6): 1 DOI: 10.5334/ijic.3003 PMC5854016 29588634

[5] WHO. Strengthening people-centred health systems in the WHO European Region: Framework for action on integrated health services delivery WHO Regional Office for Europe; 2016 Available from: http://www.euro.who.int/__data/assets/pdf_file/0004/315787/66wd15e_FFA_IHSD_160535.pdf?ua=1.

[6] Gonzalez-Ortiz, LG, Calciolari, S, Goodwin, N and Stein, V. The Core Dimensions of Integrated Care: A Literature Review to Support the Development of a Comprehensive Framework for Implementing Integrated Care. International journal of integrated care, 2018; 18(3): 10 DOI: 10.5334/ijic.4198 PMC6137610 30220893

[7] Minkman, MM. Values and Principles of Integrated Care. International journal of integrated care, 2016; 16(1): 2 DOI: 10.5334/ijic.2458 PMC5015537 27616947

[8] Haggerty, JL, Reid, RJ, Freeman, GK, Starfield, BH, Adair, CE and McKendry, R. Continuity of care: A multidisciplinary review. BMJ (Clinical research ed), 2003; 327(7425): 1219–21. DOI: 10.1136/bmj.327.7425.1219 14630762 PMC274066

[9] Bentler, SE, Morgan, RO, Virnig, BA and Wolinsky, FD. The association of longitudinal and interpersonal continuity of care with emergency department use, hospitalization, and mortality among Medicare beneficiaries. PloS one, 2014; 9(12): e115088 DOI: 10.1371/journal.pone.0115088 25531108 PMC4274086

[10] van Walraven, C, Oake, N, Jennings, A and Forster, AJ. The association between continuity of care and outcomes: A systematic and critical review. Journal of evaluation in clinical practice, 2010; 16(5): 947–56. DOI: 10.1111/j.1365-2753.2009.01235.x 20553366

[11] Zonneveld, N, Driessen, N, Stussgen, RAJ and Minkman, MMN. Values of Integrated Care: A Systematic Review. International journal of integrated care, 2018; 18(4): 9 DOI: 10.5334/ijic.4172 PMC6251066 30498405

[12] Gibson, PG, Powell, H, Coughlan, J, Wilson, AJ, Abramson, M, Haywood, P, et al. Self-management education and regular practitioner review for adults with asthma. The Cochrane database of systematic reviews, 2003; 1: Cd001117 DOI: 10.1002/14651858.CD001117 12535399

[13] Robert Wood Johnson Foundation ICIC. http://www.improvingchroniccare.org. Accessed November 20, 2005.

[14] Crossing the Quality Chasm: A New Health System for the 21st Century Washington, DC: National Academy Press; 2001. 25057539

[15] Berwick, DM, Nolan, TW and Whittington, J. The triple aim: Care, health, and cost. Health affairs, (Project Hope), 2008; 27(3): 759–69. DOI: 10.1377/hlthaff.27.3.759 18474969

[16] Wadmann, S, Strandberg-Larsen, M and Vrangbaek, K. Coordination between primary and secondary healthcare in Denmark and Sweden. International journal of integrated care, 2009; 9: e04 DOI: 10.5334/ijic.302 19340328 PMC2663705

[17] Ahgren, B and Axelsson, R. A decade of integration and collaboration: The development of integrated health care in Sweden 2000–2010. International journal of integrated care, 2011; 11 Spec Ed: e007 DOI: 10.5334/ijic.566 21677844 PMC3111884

[18] Swedish National Board of Health and Welfare S. Samverkan vid in och utskrivning av patienter i sluten vård (Cooperation in connection with registration and discharge of patients in hospital care). Available from: http://www.socialstyrelsen.se/. Accessed 180111.

[19] Wibe, T, Ekstedt, M and Helleso, R. Information practices of health care professionals related to patient discharge from hospital. Informatics for health & social care, 2015; 40(3): 198–209. DOI: 10.3109/17538157.2013.879150 24475936

[20] Berglund, H, Blomberg, S, Duner, A and Kjellgren, K. Organizing integrated care for older persons: Strategies in Sweden during the past decade. Journal of health organization and management, 2015; 29(1): 128–51. DOI: 10.1108/JHOM-04-2013-0082 25735557

[21] SFS 2014:821. Act concerning patients Sweden: Ministry of Health and Social Affairs.

[22] Tshiananga, JK, Kocher, S, Weber, C, Erny-Albrecht, K, Berndt, K and Neeser, K. The effect of nurse-led diabetes self-management education on glycosylated hemoglobin and cardiovascular risk factors: A meta-analysis. The Diabetes educator, 2012; 38(1): 108–23. DOI: 10.1177/0145721711423978 22116473

[23] Moser, A, van der Bruggen, H and Widdershoven, G. Competency in shaping one’s life: Autonomy of people with type 2 diabetes mellitus in a nurse-led, shared-care setting; a qualitative study. International journal of nursing studies, 2006; 43(4): 417–27. DOI: 10.1016/j.ijnurstu.2005.06.003 16112674

[24] Ovretveit, J, Hansson, J and Brommels, M. An integrated health and social care organisation in Sweden: Creation and structure of a unique local public health and social care system. Health policy (Amsterdam, Netherlands), 2010; 97(2–3): 113–21. DOI: 10.1016/j.healthpol.2010.05.012 20557972

[25] https://www.riksdagen.se/sv/dokument-lagar/dokument/svensk-forfattningssamling/lag-2008962-om-valfrihetssystem_sfs-2008-962.

[26] Patton, MQ. Qualitative research and evaluation methods Thousands Oaks, London and New Dehli: Sage; 2002.

[27] Merton, RK, Kendall, PL and Fiske, M. The focused interview: A manual of problems and procedures New York: Free Pree; 1990.

[28] Morgan, DL. Focus groups as qualitative research Thousand Oaks, Calif.: Sage; 1997 DOI: 10.4135/9781412984287

[29] Graneheim, UH and Lundman, B. Qualitative content analysis in nursing research: Concepts, procedures and measures to achieve trustworthiness. Nurse education today, 2004; 24(2): 105–12. DOI: 10.1016/j.nedt.2003.10.001 14769454

[30] Graneheim, UH, Lindgren, BM and Lundman, B. Methodological challenges in qualitative content analysis: A discussion paper. Nurse education today, 2017; 56: 29–34. DOI: 10.1016/j.nedt.2017.06.002 28651100

[31] Schreier, M. Qualitative content analysis in practice Thousand Oaks, CA: Sage; 2012.

[32] WMA (World Medical Association Declaration of Helsinki). Ethical Principles for Medical Research Involving Human Subjects Seoul: 59th WMA General Assembly; 2008 https://www.wma.net/policies-post/wma-declaration-of-helsinki-ethical-principles-for-medical-research-involving-human-subjects/ DOI: 10.1515/9783110208856.233

[33] All European Academies (ALLEA). Permanent Working Group on Science and Ethics. Statement on ethics education in science; 2013 Retrieved September 9, 2014 from http://www.allea.org/Content/ALLEA/Statement_Ethics_Edu_web_final_2013_10_10.pdf. 2013.

[34] Valentijn, PP, Schepman, SM, Opheij, W and Bruijnzeels, MA. Understanding integrated care: A comprehensive conceptual framework based on the integrative functions of primary care. International journal of integrated care, 2013; 13: e010 DOI: 10.5334/ijic.886 23687482 PMC3653278

[35] Ross, SE and Lin, CT. The effects of promoting patient access to medical records: A review. Journal of the American Medical Informatics Association: JAMIA, 2003; 10(2): 129–38. DOI: 10.1197/jamia.M1147 12595402 PMC150366

[36] Wibe, T, Helleso, R, Slaughter, L and Ekstedt, M. Lay people’s experiences with reading their medical record. Social science & medicine, 2011; 72(9): 1570–3. DOI: 10.1016/j.socscimed.2011.03.006 21497971

[37] McEwen, MM, Baird, M, Pasvogel, A and Gallegos, G. Health-illness transition experiences among Mexican immigrant women with diabetes. Family & community health, 2007; 30(3): 201–12. DOI: 10.1097/01.FCH.0000277763.70031.0d 17563482

[38] Inzucchi, SE, Bergenstal, RM, Buse, JB, Diamant, M, Ferrannini, E, Nauck, M, et al. Management of hyperglycaemia in type 2 diabetes: A patient-centered approach. Position statement of the American Diabetes Association (ADA) and the European Association for the Study of Diabetes (EASD). Diabetologia, 2012; 55(6): 1577–96. DOI: 10.1007/s00125-012-2534-0 22526604

[39] Rasmussen, B, O’Connell, B, Dunning, P and Cox, H. Young women with type 1 diabetes’ management of turning points and transitions. Qualitative health research, 2007; 17(3): 300–10. DOI: 10.1177/1049732306298631 17301339

[40] Daker-White, G, Hays, R, McSharry, J, Giles, S, Cheraghi-Sohi, S, Rhodes, P, et al. Blame the Patient, Blame the Doctor or Blame the System? A Meta-Synthesis of Qualitative Studies of Patient Safety in Primary Care. PloS one, 2015; 10(8): e0128329 DOI: 10.1371/journal.pone.0128329 26244494 PMC4526558

[41] SFS 2008:962. Lagen om valfrihetssystem (LOV). Act on free choice systems Stockholm: Swedish Government.

[42] Axelsson, R and Axelsson, SB. Integration and collaboration in public health–a conceptual framework. The International journal of health planning and management, 2006; 21(1): 75–88. DOI: 10.1002/hpm.826 16604850

[43] Gulliford, M, Naithani, S and Morgan, M. What is ‘continuity of care’? Journal of health services research & policy, 2006; 11(4): 248–50. DOI: 10.1258/135581906778476490 17018200

